# A comparative study of time series foundation models for hand, foot, and mouth disease forecasting: TimesFM, Moirai, and traditional approaches

**DOI:** 10.3389/fpubh.2025.1634138

**Published:** 2025-09-25

**Authors:** Yanqin Wang, Guoyu Huang, Ceran Chen, Qiu Li, Ximing Xu

**Affiliations:** ^1^Chongqing Key Laboratory of Childhood Nutrition and Health, Department of Nephrology, Children's Hospital of Chongqing Medical University, National Clinical Research Center for Child Health and Disorders, Ministry of Education Key Laboratory of Child Development and Disorders, China International Science and Technology Cooperation Base of Child Development and Critical Disorders, Chongqing, China; ^2^Big Data Center for Children's Medical Care, Children's Hospital of Chongqing Medical University, Chongqing, China

**Keywords:** HFMD, time series analysis, foundation models, epidemic prediction, epidemiological modeling

## Abstract

**Background:**

Hand, foot, and mouth disease (HFMD) is a pediatric infectious disease prevalent in the Asia-Pacific region, requiring accurate forecasting for effective public health interventions. This study aims to compare the performance of time series foundation models (TimesFM and Moirai) with traditional methods (ARIMA and LSTM) in predicting HFMD outbreaks across various datasets and forecasting horizons.

**Methods:**

The study analyzed weekly HFMD incidence data from Korea (2015–2024), Singapore (2012–2018), and Chongqing, China (2015–2024). Zero-shot versions of TimesFM (200 M and 500 M) and Moirai models were assessed against ARIMA and LSTM using forecasting horizons of 1 week, 5 weeks, and 10 weeks. Lookback windows of 50 and 100 weeks were used across experiments. Performance was evaluated based on forecasting accuracy across all datasets. Computational resource requirements were also analyzed.

**Results:**

For 1-step predictions, ARIMA and Moirai delivered comparable results. TimesFM-500 M achieved the best performance for 5-step predictions with 100-week lookback windows across all datasets. For 10-step predictions, TimesFM-200 M performed well with 50-week lookback windows but showed weaker results with longer historical data. Foundation models demonstrated the potential for robust HFMD forecasting but required greater computational resources.

**Conclusion:**

Time series foundation models can effectively predict HFMD outbreaks. While these models require more computational resources, their zero-shot capabilities simplify the forecasting process by eliminating the need for retraining.

## 1 Introduction

Hand, foot, and mouth disease (HFMD) is a common infectious disease that primarily affects children under 5 years of age and is caused by human enterovirus infections (1). Typical symptoms include fever, oral sores, and rashes on hands and feet. While most patients present mild symptoms and recover naturally within a week, some severe cases can lead to life-threatening complications (2). Over the past two decades, HFMD has caused multiple outbreaks worldwide, particularly in the Asia–Pacific region (3, 4). Accurate forecasting models are critical for improving disease surveillance, guiding medical resource allocation, and supporting targeted prevention strategies to mitigate public health risks (5, 6).

Autoregressive integrated moving average (ARIMA) and long short-term memory (LSTM) are traditional models widely used in epidemiological forecasting, including HFMD prediction in many countries and regions (7–10). However, ARIMA has basic limitations in capturing complex non-linear dynamics and long-term dependencies (11). LSTM networks overcome these limitations through their gated architecture, selectively retaining or forgetting information over long time sequences (12). This makes them excel at handling long sequence data and capturing long-term dependencies, and they have also shown good performance in epidemiological applications (13, 14). Nevertheless, LSTM models require extensive training data and computational resources for training (15, 16).

Recently, researchers have developed multiple time series foundation models (TSFMs) for time series analysis. These models use large-scale cross-domain pretraining to extract universal features for complex and heterogeneous prediction problems. TSFMs function as foundational building blocks for forecasting, classification, anomaly detection, and imputation. They offer effective out-of-the-box performance with minimal data requirements and can be fine-tuned for enhanced performance (17). Among these models, the masked encoder-based universal time series forecasting transformer (Moirai) from Salesforce and the time series foundation model (TimesFM) from Google are two representative models in terms of their architectural design, open-source availability, and flexibility of use. Moirai, which is based on a masked encoder architecture, learns universal time series features through pretraining on the LOTSA dataset. Its training objective is to reconstruct randomly masked segments of time series, enabling it to capture both the global context and local temporal patterns. The LOTSA dataset includes 27 billion observations across nine domains, such as healthcare, meteorology, economics, and transportation, including the COVID-19 time series. The pretraining objective of Moirai is to reconstruct randomly masked time series segments, enabling it to capture both global context and local temporal features, which are particularly important when dealing with heterogeneous and noisy data distributions like HFMD outbreaks (18). TimesFM adopts an autoregressive decoding structure with longer output patches, learning temporal patterns and contextual relationships through future sequence generation during pre-training. It supports dynamic prediction and handles long sequence generation, particularly in zero-shot and few-shot learning scenarios (19).

However, these TSFMs have not been applied to HFMD prediction. In this study, we evaluate the performance of these time series foundation models in HFMD forecasting and compare them with ARIMA and LSTM.

## 2 Methods

### 2.1 Data collection and study design

This study analyzes HFMD data collected from three regions in Asia. Data from Korea were obtained from the Korea Disease Control and Prevention Agency (KDCA) as publicly available weekly case counts (20). These data were collected by the KDCA through a well-established national reporting system in collaboration with designated surveillance institutions. Singaporean data were obtained from weekly reports published on the Singapore government's open data platform and were collected through healthcare reports, laboratory confirmations, and community-based monitoring programs. We excluded Singapore data after December 2018 because negligible HFMD cases were reported between January 2019 and December 2022 (21). For China, we obtained data from the Children's Hospital of Chongqing Medical University, covering the period from January 2015 to December 2024 (22). These data were collected following standardized hospital reporting protocols, and the original daily case counts from the hospital were aggregated into weekly totals for analysis. All datasets, accessed on January 10, 2025, consisted exclusively of aggregate case counts without any demographic or personally identifiable information.

The last 100 time points of each dataset were designated the test set to evaluate model performance. Comparative experiments were conducted across forecasting horizons of 1, 5, and 10 steps. For the LSTM, TimesFM, and Moirai models, performance comparisons were made under different lookback window lengths of 50 and 100.

### 2.2 ARIMA

In the ARIMA (p, d, q) model, parameter p denotes the order of autoregression, d represents the degree of differencing, and q indicates the order of the moving average. The appropriate (p, d, q) parameters are determined via the Akaike information criterion (AIC). For multistep forecasting, the model employs an iterative approach where single-step predictions are recursively fed as inputs until they reach the target forecast horizons. For all tested horizons (1-step, 5-step, and 10-step), the identified ARIMA parameters are 5,0,0 for the Korean dataset 5,0,2 for the Singapore dataset, and 1,1,0 for the CHCMU dataset.

### 2.3 LSTM

The LSTM model constructed in this study adopts a two-layer hidden layer structure, with each layer configured with 100-dimensional hidden units, and uses the GELU as the activation function. In terms of the training configuration, the model's batch size is 32, and the Adam optimizer with a learning rate of 0.001 is used. Unlike ARIMA models, which require iterative prediction, this LSTM architecture can directly output prediction sequences for multiple time steps (h=5 and h=10) through a single forward computation.

### 2.4 TimesFM and Moirai

This study used two sizes of TimesFM (TimesFM-1.0–200 M and TimesFM-2.0–500 M) (19) and three sizes of Moirai (Moirai-Small, Moirai-Base, and Moirai-Large with 14 M, 91 M, and 311 M parameters, respectively) (18). The input sequences were processed by sliding windows of 50 and 100 weeks. All the models were operated in zero-shot mode without additional fine-tuning on our datasets.

### 2.5 Evaluation of model performance

To assess the model's performance thoroughly, this study uses the root mean square error (RMSE) and mean absolute error (MAE). These metrics evaluate the model's performance from different perspectives. The formulas for calculating these metrics are provided.


$$\begin{array}{l} RMSE=\sqrt{\frac{1}{n}\sum_{i=1}^{n}{\left({\hat{y}}_{i}-y_{i}\right)}^{2}}\\ \text{ M}AE=\frac{1}{n}\sum_{i=1}^{n}\left|{\hat{y}}_{i}-y_{i}\right| \end{array}$$

where *y*_*i*_ is the actual value at the i-th time point, ŷ_*i*_ is the predicted value at the i-th time point, and ${\hat{y}}_{i}$ is the mean value of the actual values.

### 2.6 Model training setups

The training hardware configuration includes an NVIDIA RTX 4090 GPU and 24 GB of memory, with all the models trained on the same server environment. The software environment includes Python 3.10, which uses main libraries such as Sklearn, Statsmodels for ARIMA, and PyTorch for the LSTM, TimesFM and Moirai models.

## 3 Results

### 3.1 The datasets

**Figure 1:**
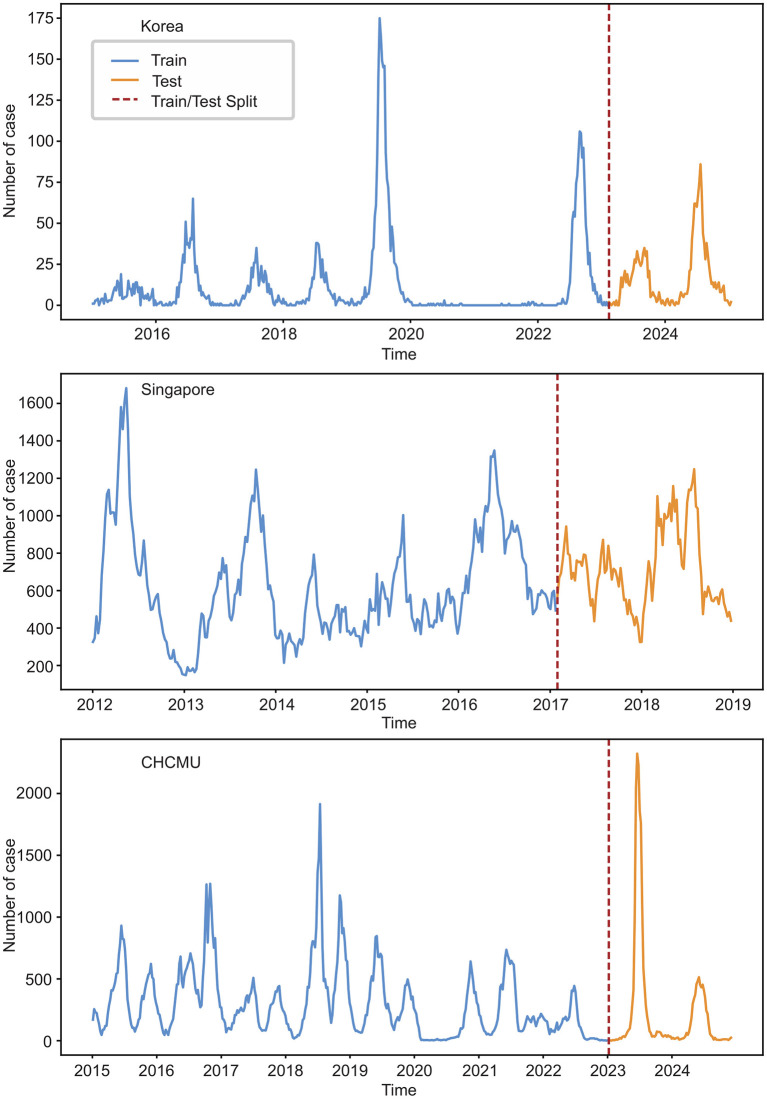
Weekly HFMD cases in Korea, Singapore, and the CHCMU.

The Korean dataset contains 524 data points, with an incidence of 12.41 ± 23.71 cases per week. The Singapore dataset consists of 365 data points, with an incidence of 644.96 ± 273.29 cases per week. The Chinese CHCMU dataset includes 518 data points, with an incidence of 282.43 ± 324.67 cases per week (Figure 1).

### 3.2 Single-step forecasting

For single-step prediction tasks, when the lookback window is set to 50 weeks, both ARIMA and Moirai achieve excellent performance across all three datasets with comparable prediction accuracies. Specifically, on the Korean dataset, the ARIMA model achieves an MAE of 4.286 and an RMSE of 6.193, whereas Moirai-Base shows similar performance, with an MAE of 4.303 and an RMSE of 6.302. For the CHCMU dataset, Moirai-Base attains an MAE of 48.763 and an RMSE of 129.987, approaching the performance of the ARIMA model, with an MAE of 50.068 and an RMSE of 124.427.

When the lookback window is extended to 100 weeks, for the Korean dataset, Moirai-Base records the lowest MAE (4.266), whereas TimesFM-500 M obtains the lowest RMSE (5.980). For the Singapore dataset, ARIMA performs best, with both the lowest MAE (79.298) and RMSE (99.901). For the CHCMU dataset, TimesFM-500 M records the lowest MAE (47.866), whereas ARIMA maintains the lowest RMSE (124.427). The prediction capability of the TimesFM models tends to improve as the lookback window length increases (Table 1). Visual comparisons of performance across all prediction steps and models are plotted in Supplementary Figure S1.

**Table 1:** Comparison of model performance in HFMD forecasting across Korea, Singapore, and CHCMU (1 week step).

**Data**	**Model**	**50 weeks' lookback**		**100 weeks' lookback**	
		**MAE**	**RMSE**	**MAE**	**RMSE**
Korea	ARIMA	4.286	6.193	4.286	6.193
	LSTM	5.385	7.366	6.084	8.429
	TimesFM-200M	4.619	6.910	4.440	6.866
	TimesFM-500M	4.415	6.620	4.305	5.980
	Moirai-14M	4.386	6.338	4.433	6.282
	Moirai-91M	4.303	6.302	4.266	6.181
	Moirai-311M	4.375	6.413	4.328	5.992
Singapore	ARIMA	79.298	99.901	79.298	99.901
	LSTM	104.736	132.397	115.750	151.468
	TimesFM-200M	84.714	107.631	80.768	101.666
	TimesFM-500M	83.991	105.630	81.314	101.735
	Moirai-14M	78.475	101.293	80.466	101.168
	Moirai-91M	84.506	104.254	87.144	107.038
	Moirai-311M	83.027	103.882	83.884	104.380
CHCMU	ARIMA	50.068	124.427	50.068	124.427
	LSTM	53.217	145.046	55.106	155.126
	TimesFM-200M	76.496	202.875	65.939	160.085
	TimesFM-500M	59.811	156.116	47.866	132.966
	Moirai-14M	78.216	231.271	80.254	228.189
	Moirai-91M	48.763	129.987	50.105	128.121
	Moirai-311M	56.170	168.349	61.582	171.215

The ARIMA model is evaluated only once per dataset, as its performance is not dependent on varying lookback periods due to its inherent model structure.

### 3.3 Five-step forecasting

For five-step prediction tasks, with a lookback window of 50 weeks, TimesFM-500 M achieves the best results on the Singapore dataset, with an MAE of 121.498 and an RMSE of 165.370, closely followed by ARIMA, with an MAE of 122.199 and an RMSE of 166.696. For the CHCMU dataset, TimesFM-200 M achieves the best performance, with an MAE of 121.026 and an RMSE of 349.123.

When the lookback window extends to 100 weeks, TimesFM-500 M shows significant improvement, achieving the highest prediction accuracy across all three datasets. For the Korean dataset, the MAE decreases from 7.201 to 6.252, a reduction of 13.18%, and the RMSE decreases from 67.01 to 60.53, a decrease of 9.69%. For the CHCMU dataset, the MAE decreases from 121.026 to 101.153, a decrease of 16.22%, whereas the RMSE improves from 349.123 to 269.97, a decrease of 22.62%. These results indicate that the prediction capability of TimesFM-500 M substantially improves with increasing lookback window length (Table 2).

**Table 2:** Comparison of model performance in HFMD forecasting across Korea, Singapore, and the CHCMU (5-week step).

**Data**	**Model**	**50 weeks' lookback**		**100 weeks' lookback**	
		**MAE**	**RMSE**	**MAE**	**RMSE**
Korea	ARIMA	7.780	11.016	7.780	11.016
	LSTM	7.029	9.806	7.675	10.858
	TimesFM-200M	7.468	11.571	7.297	12.000
	TimesFM-500M	7.201	11.500	6.252	8.898
	Moirai-14M	7.278	12.292	7.478	12.225
	Moirai-91M	7.455	12.842	7.140	12.075
	Moirai-311M	7.234	12.233	7.578	12.300
Singapore	ARIMA	122.199	166.696	122.199	166.696
	LSTM	136.965	184.293	161.713	205.639
	TimesFM-200M	134.964	177.035	123.382	164.551
	TimesFM-500M	121.498	165.370	121.992	157.212
	Moirai-14M	127.477	170.460	128.617	171.341
	Moirai-91M	131.355	177.888	131.467	174.574
	Moirai-311M	134.001	182.264	129.854	172.836
CHCMU	ARIMA	174.606	411.755	174.606	411.755
	LSTM	161.381	347.738	181.625	396.302
	TimesFM-200M	121.026	349.123	159.234	415.901
	TimesFM-500M	161.570	413.919	135.358	373.807
	Moirai-14M	174.815	430.278	164.678	409.655
	Moirai-91M	151.215	372.112	153.515	376.545
	Moirai-311M	152.351	398.606	153.981	393.631

### 3.4 Ten-step forecasting

For ten-step prediction tasks with a 50-week lookback window, TimesFM-200 M achieves the best overall performance. For the Korean dataset, it achieves an MAE of 7.344 and an RMSE of 11.503. For the CHCMU dataset, the lowest MAE of 154.640 is recorded. For the Singapore dataset, TimesFM-200 M has an MAE of 160.263 and an RMSE of 209.113, which are slightly higher than the performance of LSTM, with an MAE of 156.755 and an RMSE of 200.940. Unlike single-step and five-step predictions, when the lookback window increases to 100 weeks, all the TimesFM models show a decline in performance (Table 3).

**Table 3:** Comparison of model performance in HFMD forecasting across Korea, Singapore, and the CHCMU (10-week step).

**Data**	**Model**	**50 weeks' lookback**		**100 weeks' lookback**	
		**MAE**	**RMSE**	**MAE**	**RMSE**
Korea	ARIMA	8.967	12.132	8.967	12.132
	LSTM	9.211	12.039	10.077	12.882
	TimesFM-200M	7.344	11.503	9.164	13.933
	TimesFM-500M	9.464	14.025	10.418	14.480
	Moirai-14M	9.573	13.916	9.632	13.913
	Moirai-91M	9.764	14.363	9.666	13.571
	Moirai-311M	9.617	14.320	9.753	14.030
Singapore	ARIMA	189.051	243.423	189.051	243.423
	LSTM	156.755	200.940	156.007	196.084
	TimesFM-200M	160.263	209.113	165.498	201.836
	TimesFM-500M	164.485	222.534	182.598	226.180
	Moirai-14M	173.156	231.400	186.730	236.480
	Moirai-91M	171.767	239.127	185.645	245.123
	Moirai-311M	178.478	244.470	177.581	238.388
CHCMU	ARIMA	190.854	391.950	190.854	391.950
	LSTM	200.285	395.594	194.871	397.249
	TimesFM-200M	154.640	398.468	234.949	517.419
	TimesFM-500M	155.059	402.234	196.644	452.071
	Moirai-14M	183.507	390.850	171.293	386.361
	Moirai-91M	168.394	363.673	164.136	360.974
	Moirai-311M	163.601	385.678	167.007	389.357

## 4 Discussion

This study presents the first evaluation of TSFMs for predicting the incidence of HFMD, comparing the performance of TimesFM and Moirai with that of traditional models (ARIMA and LSTM). Using weekly incidence data from Korea, Singapore, and Chongqing, China, the comparison examines different temporal scales for both prediction horizons and historical windows. The findings reveal that for single-step prediction, ARIMA and Moirai achieve comparable and excellent performance. For five-step prediction with a 100-week lookback window, TimesFM-500 M demonstrates superior performance across all three datasets. For ten-step prediction, TimesFM-200 M performs well but does not benefit from increased historical windows.

Foundation time series models outperform traditional approaches across multiple prediction settings without task-specific fine-tuning. This is attributed to their ability to leverage large-scale pretraining on diverse datasets and advanced architectures, such as Moirai's multi-scale projection and TimesFM's patch-based decoding, which enhance their capacity to model temporal patterns and adapt to geographic variability (23–25). Emerging research suggests that TSFM performance improves with increases in model scale, data diversity, and computational resources, highlighting these factors as key to their predictive success across varied time-series scenarios (26). However, traditional models such as ARIMA remain highly effective in specific use cases, indicating that model selection should be context dependent.

While time series foundation models require more computational resources for training and inference, they offer the advantage of zero-shot prediction, which eliminates the need for retraining on new datasets and reduces long-term costs (18). In contrast, traditional methods use less computational power but need expertise to determine the best parameters and architectures for each specific task and dataset. Thus, in cases where computational resources are available and quick deployment is needed, pre-trained models may be the better choice.

The performance differences between TimesFM and Moirai may be related to their architectural designs. TimesFM's decoder-only architecture appears to benefit long-term prediction tasks, possibly because of its ability to generate predictions incrementally and adjust them on the basis of previous predictions. This characteristic in handling temporal dynamics might contribute to improved long-term prediction accuracy (27), which matches our observations of relatively better performance in medium- to long-term predictions.

This study has several limitations. The geographical focus is narrow, and validation was limited to HFMD, which affects the generalizability of the findings. Uncertainty quantification was not explicitly addressed, and model performance under operational constraints, such as limited computational resources, was not tested. Further work should include fine-tuning for specific tasks to optimize pre-trained model performance.

In summary, this study explores the potential of time series foundation models in predicting the incidence of HFMD. The demonstrated zero-shot prediction capabilities and relatively better performance in certain settings offer new technical options for HFMD warning systems, with implications for enhancing disease surveillance and informing epidemic prevention strategies. However, its applicability to other infectious diseases remains to be validated. Future studies could improve prediction accuracy and broaden applicability by refining model architecture, validating against other diseases, and incorporating multisource data alongside epidemiological data to better understand the dynamics of disease transmission.

## Data Availability

Publicly available datasets were analyzed in this study. This data can be found here: The Korean HFMD data are publicly accessible from the Korea Disease Control and Prevention Agency (KDCA) Infectious Disease Portal (https://dportal.kdca.go.kr/pot/is/st/hfmd.do). Singapore HFMD data are available via Singapore's open data platform (https://data.gov.sg/datasets?agencies=Ministry+of+Health+(MOH)&amp;page=1&amp;query=infectious&amp;resultId=d_ca168b2cb763640d72c4600a68f9909e). Data from Children's Hospital of Chongqing Medical University are available upon reasonable request by contacting the corresponding author.
