# Supplementary material for: A comparative study of time series foundation models for hand, foot, and mouth disease forecasting: TimesFM, Moirai, and traditional approaches
Source: Front Public Health. 2025 Sep 25;13:1634138. doi: 10.3389/fpubh.2025.1634138 (PMC12507834; doi:10.3389/fpubh.2025.1634138)

Supplementary Material

Supplementary Figure S1. Comparison of Model Performance across Different Prediction Steps and Lookback Windows in Three Regions.

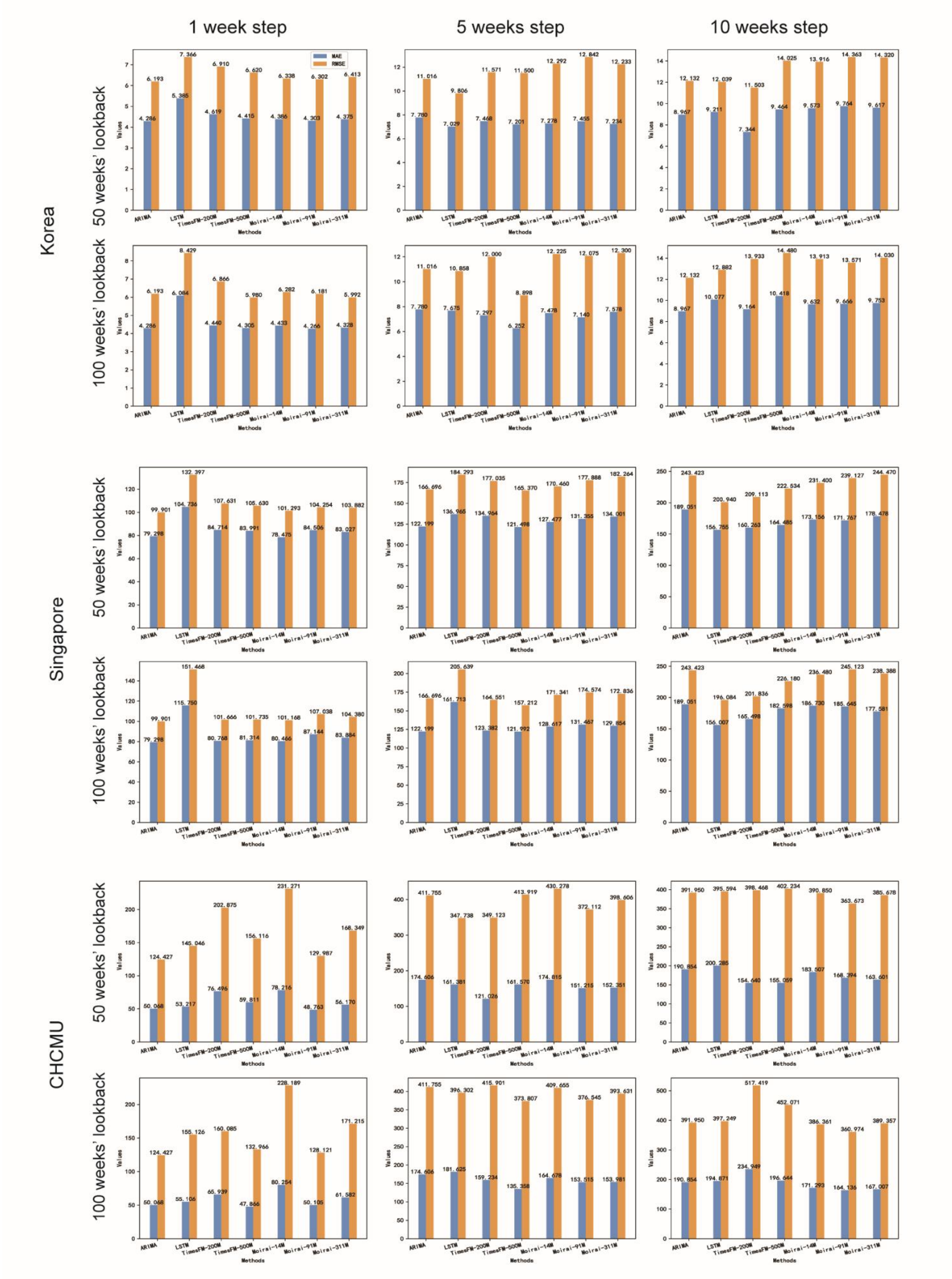

Supplement: Supplementary file 1 [file Data_Sheet_1.pdf]
